# Accuracy of dengue clinical diagnosis with and without NS1 antigen rapid test: Comparison between human and Bayesian network model decision

**DOI:** 10.1371/journal.pntd.0006573

**Published:** 2018-06-18

**Authors:** Chaitawat Sa-ngamuang, Peter Haddawy, Viravarn Luvira, Watcharapong Piyaphanee, Sopon Iamsirithaworn, Saranath Lawpoolsri

**Affiliations:** 1 Department of Tropical Hygiene, Faculty of Tropical Medicine, Mahidol University, Bangkok, Thailand; 2 Faculty of Information and Communication Technology, Mahidol University, Nakhon Pathom, Thailand; 3 Department of Clinical Tropical Medicine, Faculty of Tropical Medicine, Mahidol University, Bangkok, Thailand; 4 Department of Disease Control, Ministry of Public Health, Nonthaburi, Thailand; London School of Hygiene & Tropical Medicine, UNITED KINGDOM

## Abstract

Differentiating dengue patients from other acute febrile illness patients is a great challenge among physicians. Several dengue diagnosis methods are recommended by WHO. The application of specific laboratory tests is still limited due to high cost, lack of equipment, and uncertain validity. Therefore, clinical diagnosis remains a common practice especially in resource limited settings. Bayesian networks have been shown to be a useful tool for diagnostic decision support. This study aimed to construct Bayesian network models using basic demographic, clinical, and laboratory profiles of acute febrile illness patients to diagnose dengue. Data of 397 acute undifferentiated febrile illness patients who visited the fever clinic of the Bangkok Hospital for Tropical Diseases, Thailand, were used for model construction and validation. The two best final models were selected: one with and one without NS1 rapid test result. The diagnostic accuracy of the models was compared with that of physicians on the same set of patients. The Bayesian network models provided good diagnostic accuracy of dengue infection, with ROC AUC of 0.80 and 0.75 for models with and without NS1 rapid test result, respectively. The models had approximately 80% specificity and 70% sensitivity, similar to the diagnostic accuracy of the hospital’s fellows in infectious disease. Including information on NS1 rapid test improved the specificity, but reduced the sensitivity, both in model and physician diagnoses. The Bayesian network model developed in this study could be useful to assist physicians in diagnosing dengue, particularly in regions where experienced physicians and laboratory confirmation tests are limited.

## Author summary

In many parts of the world, dengue diagnosis still relies on clinical manifestation and basic laboratory tests. However, clinical manifestation of dengue patients is usually unspecific especially during the early stage, which makes accurate dengue diagnosis a challenge for physicians. Recently, the World Health Organization has recommended the use of the NS1 antigen rapid test to assist in dengue diagnosis. Unfortunately, the rapid test is still only in limited use due to its cost and relatively low sensitivity. In this study we constructed Bayesian Network (BN) models from available data and used them to predict the probability of dengue infection. Our BN models provided good performance in making dengue diagnosis, comparable with physician’s diagnosis. Information on NS1 rapid test could improve the diagnostic performance by improving specificity of dengue diagnosis. This study suggested that BN models can be useful to assist physicians in dengue diagnosis, particularly in areas where experienced physicians and laboratory confirmation tests are limited.

## Introduction

Dengue is considered a global public health threat due to its potential to rapidly spread across countries [1]. Currently, dengue is endemic in more than 100 countries, with approximately 2.5 billion people at risk. [2, 3]. Dengue infections lead to a wide range of clinical outcomes. The large proportion of asymptomatic and mild dengue infections can lead to underreporting of dengue cases by routine passive surveillance systems [4–6].

Due to unspecific clinical manifestation during the early phase of dengue infection, differentiating dengue patients from other acute febrile illness cases is a great challenge. Clinical diagnosis alone may lead to misdiagnosis which can subsequently lead to serious clinical outcome [7, 8]. Several laboratory confirmation tests for dengue infection are currently available, including serological test, virus detection, antigen detection, and genome detection. The use of these tests is still limited since they require sophisticated equipment and well-trained staff [9–11]. The accuracy of these tests may vary over the course of illness [12]. Since the serological test requires a pair of serum samples taken two weeks apart to confirm a rise in IgM/IgG dengue antibody, this test may not be practical for initial diagnosis of dengue [12]. Other factors could also interfere with the interpretation of the immunological assays, such as history of immunization or previous infection by dengue or other flavivirus. Virus and genome detection can directly identify dengue viral infection. However, virus detection must be performed during the viremic stage, within 1–5 days of onset of fever [1].

Recently, the World Health Organization (WHO) has recommended the use of a rapid test to detect dengue NS1 antigen [1], as it is simple to use and can provide results within 30 minutes [13]. The accuracy of the NS1 antigen rapid test is considered high with sensitivity 55%-82% and specificity 97%-100%. Since the NS1 rapid test aims to detect dengue NS1 antigen, it should be performed within 5 days of onset of fever. However, this rapid test is not generally performed in many health facilities due to its cost [1]. Even though laboratory confirmation is highly recommended for dengue diagnosis, laboratory tests for dengue infection are generally inaccessible, particularly in resource limited settings. Commonly, physicians make diagnosis of dengue infection based only on clinical signs and symptoms [2], even though clinical diagnosis is difficult and may vary, particularly among inexperienced physicians [14, 15].

Application of machine learning techniques in health practice has been shown to speed up the process of diagnosis, and help avoid some human errors [16]. Bayesian networks (BN) are one such approach and have been widely applied in medicine due to their ability to effectively handle uncertain information. A Bayesian network is a graphical representation of probability distribution in which nodes represent random variables and links represent direct probabilistic influence among the variables [17]. The relation between a node and its parents is quantified by a conditional probability table (CPT), specifying the probability of the random variable conditioned on all combinations of the values of the parents. The structure of the network encodes information about probabilistic independence such that the CPTs along with the independence relations provide a full specification of the joint probability distribution over the random variables represented by nodes. By decomposing a joint probability distribution into a collection of smaller local distributions (the CPTs), a Bayesian network provides a highly compact representation of the complete joint distribution. In BN diagnostic models, factors that can influence the diagnosis, including clinical signs, symptoms, and laboratory results are included in the model to form a causal relationship network [18]. Findings are entered into the model as evidence and the posterior probability of disease is computed. Previous studies have shown that Bayesian networks can accurately perform diagnosis and prediction of diseases or health conditions for wide variety of problems [19, 20].

This study aimed to construct Bayesian network models for clinical diagnosis of dengue and to compare the diagnostic accuracy of the models with that of experts. In addition, the usefulness of the NS1 rapid test to improve accuracy of dengue diagnosis was explored by comparing the accuracy of dengue diagnosis between models with and without the NS1 rapid test result. Results show that the BN models could be useful to assist physicians in dengue diagnosis, particularly in areas where laboratory resources and physician experience are limited.

## Method

### Data source

This study used secondary data collected from a previous cohort study on acute undifferentiated febrile patients conducted during March 2013 to February 2014 at the fever clinic of the Bangkok Hospital for Tropical Diseases, Faculty of Tropical Medicine, Mahidol University [21]. Patients aged > 15 years with a presence of fever less than two weeks (body temperature ≥ 37.8 C) and without specific organ infection were enrolled in the cohort. All patients in the cohort study received history taking, physical examination, basic and specific laboratory investigation for common tropical diseases, including complete blood count (CBC), blood chemistry, urinalysis, dengue NS1 rapid test, serology for dengue, PCR for dengue, blood cultures, serology for influenza, and serology and PCR for scrub typhus, murine typhus, and leptospirosis.

In addition, dengue monthly incidence in Bangkok during 2013 to 2014 was obtained from the Ministry of Public Health, Thailand. This information was used as prior knowledge that might influence a physician’s diagnosis of dengue.

### Ethics statement

Data used in this study was obtained from a cohort study previously approved by the Ethics Committee of the Faculty of Tropical Medicine, Mahidol University. In the cohort study, all adult subjects provided written informed consent, and a parent of any young subjects (15–18 years old) provided written informed consent along with the subjects. The use of the secondary data for this particular study was exempted from ethical review by the Ethics Committee of the Faculty of Tropical Medicine, Mahidol University (MUTN-EXMPT 2017–001).

### Definition of dengue infection

Dengue diagnosis in this study was made based on laboratory confirmation, following the WHO guideline. Dengue infection was defined as either positive PCR for dengue virus or a fourfold or greater change in IgG or IgM antibody titer to dengue virus antigen in paired serum samples.

### Factors related to dengue infection

Clinical signs and symptoms, physical examination including tourniquet test, and basic laboratory results at diagnosis, as well as monthly dengue incidence in Bangkok were considered as predicting factors for dengue infection. Variables considered for inclusion in the model were selected according to expert opinion, literature review, and univariate analysis of the data (Table 1).

**Table 1:** Variables considered for inclusion in the Bayesian network model.

Demographic characteristics	Clinical manifestation	Laboratory indicators
• Age • Occupation • Underlying disease • Monthly incidence report in Bangkok	• Nausea • Vomiting • Rash • Bleeding • Petechieal • Myalgia • Tourniquet test • Dehydration • Diarrhea • Day of fever	• Platelet • Hematocrit • White Blood Cell • %Lymphocyte • %Neutrophil • %Atypical lymphocyte • AST / ALT ratio • NS1 rapid test

### Bayesian network modeling

**Fig 1:**
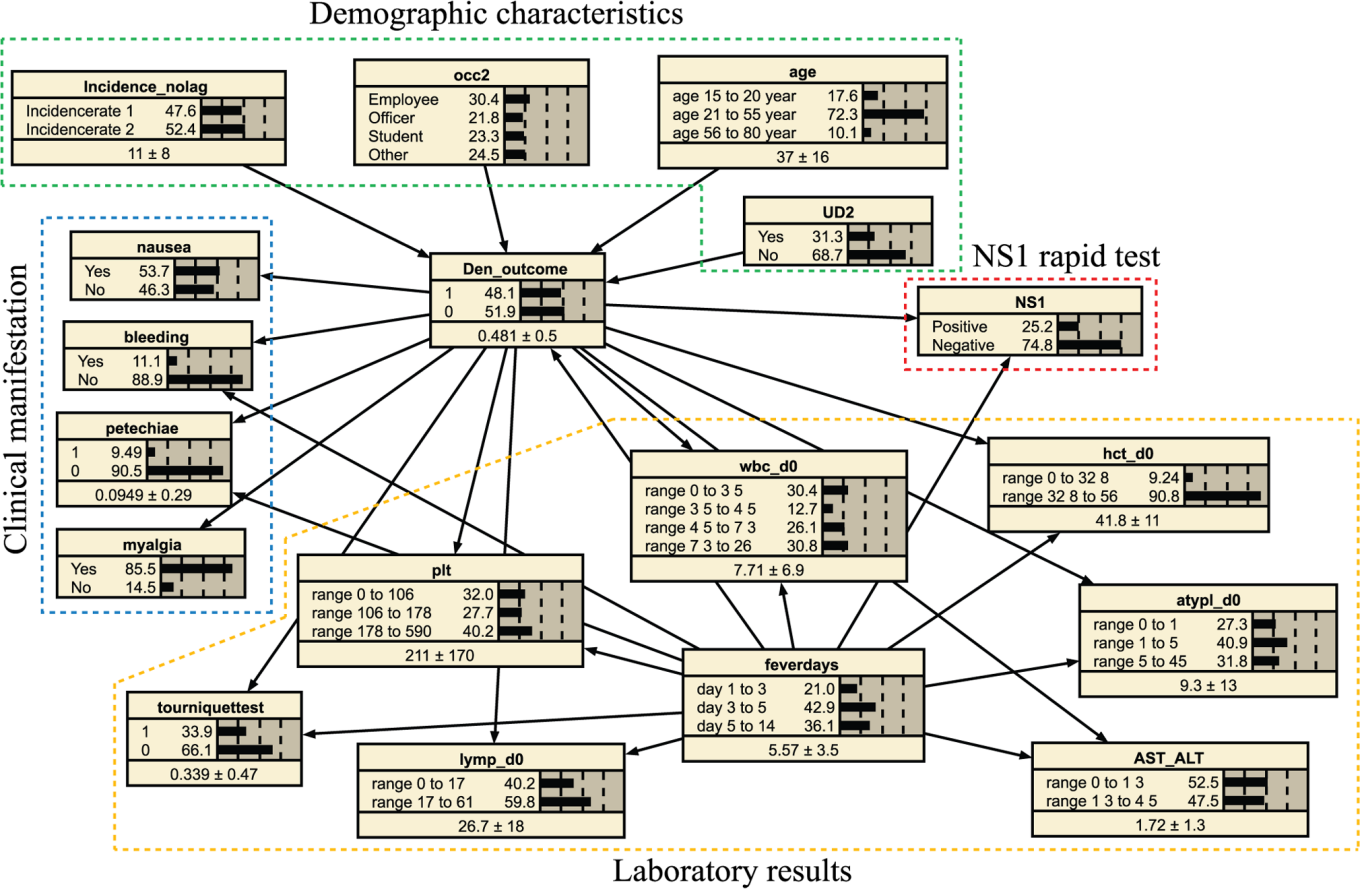
Final Bayesian network model for dengue diagnosis.

Each selected variable was represented by a node in the BN model. Links between nodes were determined based on causal relationships between predicting factors and dengue infection outcome (Fig 1). Similar to other infectious diseases, the clinical and laboratory profiles of dengue patients show a dynamic pattern over the course of disease. Therefore, duration of fever at enrollment was considered to influence all clinical and laboratory results, including the accuracy of the NS1 rapid test.

Bayesian network models were first constructed separately for demographic, clinical, and laboratory indicators. Forward selection was used to determine the optimal subset of variables for each model. This is done by iteratively introducing variables into the model one by one, at each stage selecting the variable that most improve model accuracy in terms of AUC, and stopping when no variable improves accuracy. This was used rather than backward elimination since initial inclusion of all variables in the model would result in conditional probability tables too large for the available data, which would confound attempts to determine the influence of each variable. The variables selected by forward selection on each of the three models were included in the final Bayesian network diagnostic model. Laboratory results with continuous value were discretized using the SMbinning package of R [22], where the program determined the cut-offs that provided the strongest association with dengue outcome for each value range.

The NS1 test was introduced into the model separately from the other common laboratory results as this test may not be possible to routinely perform in many hospitals. The diagnostic performance of two final BN models was determined with and without the NS1 rapid test. In addition, monthly incidence (with and without lag time) reported in Bangkok, obtained from Ministry of Public Health, was also added into the model because the current outbreak situation of dengue may influence a physician’s diagnosis.

The Netica software (Norsys Software Corp) was used to perform model construction and inference. Receiver Operating Characteristic (ROC) curve analysis was performed to determine model performance. The model with highest Area Under the Curve (AUC) value was considered the best model.

### Model validation

The models were validated using 10-fold cross validation. The data was separated into 10 sets, each set containing 10% of the data. For each validation round, 9 sets were used as training data, and the other set was used as testing data to determine diagnostic performance using the ROC analysis. After 10 rounds of validation using the different data sets, the average AUC was calculated to determine the overall performance of each model.

### Physician’s dengue diagnosis

To compare the model diagnostic performance with that of experienced physicians, two fellows in infectious disease were asked to independently review demographic, clinical and basic laboratory profiles of each patient. Two sets of patient profiles, one with NS1 rapid test results and another without NS1 test results, were given in random order to the physicians. Then the physicians were asked to make a diagnosis of either dengue infection or non-dengue infection, based on the given patient information. The Cohen-Kappa coefficient was calculated to determine the reliability of diagnosis made by the two experts. In case of inconsistent diagnosis between the two fellows, the final decision was made by a third fellow.

### Accuracy of dengue diagnosis: Comparison between BN model and physicians

The accuracy of dengue diagnosis was determined according to sensitivity, specificity, positive predictive value, and negative predictive value, considering patient profile with and without the NS1 rapid test. In the BN models, the accuracy was calculated by comparing the diagnosis by the models with the confirmed dengue infection outcome. To avoid overfitting of the models, the accuracy parameters were calculated using 10-fold cross validation datasets, in which 10% of data was used for testing model accuracy in each round. After 10 rounds of testing the different datasets, the average accuracy parameters were then determined. Similar to model accuracy estimation, the accuracy of physicians’ decisions was determined using the average of parameters estimated from the same 10-fold cross validation datasets.

## Result

### Patient characteristics

A total of 397 patients were recruited in this study, with 171(57%) of them female. Almost one fourth of the patients were students with age between 15 and 20 years. Most of patients (69%) reported that they had no underlying disease (Table 2).

**Table 2:** Demographic characteristics of acute undifferentiated febrile patients recruited in the study.

	N (%)
Total	397
Gender	
Male	226 (57)
Female	171 (43)
Age (years); Mean (SD)	33.7 (14.1)
Age groups (years)	
15–20	85 (21.4)
21–55	278 (70)
>55	34 (8.6)
Occupation	
Employee	116 (30)
Officer	90 (24)
Student	85 (22)
Others	92 (24)
Underlying disease	
Present	122 (31)
Absent	271 (69)

### Final diagnosis of patients

Among 397 acute undifferentiated febrile patients, dengue infection was confirmed in 183 (46%) patients. The majority (84%) of dengue infected cases had single infection (dengue alone), whereas the other 16% (30 cases) of dengue infected cases had co-infection with other infectious diseases such as scrub typhus, leptospirosis, influenza, murine typhus, typhoid, and hepatitis A. However, according to the statistical analysis, there was no significant difference in clinical characteristics between dengue single infection and co-infection patients.

Among the 214 non-dengue febrile patients, for more than half of them (61%) it was not possible to specifically identify causal agents. About 7%-10% of non-dengue patients had influenza, murine typhus, or leptospirosis (Table 3).

**Table 3:** Final diagnosis of dengue and non-dengue patients.

Dengue cases	N (%)	Non-dengue cases	N (%)
Dengue single infection	154 (84.1)	AUFI (Acute unidentified febrile illness)	130 (60.8)
Dengue and Leptospirosis	9 (4.9)	Murine typhus	21 (9.8)
Dengue and Influenza	8 (4.3)	Others	17 (7.9)
Dengue and Murine typhus	7 (3.8)	Leptospirosis	16 (7.5)
Dengue and Scrub typhus	2 (1.1)	Influenza	15 (7.0)
Dengue and Typhoid	1 (0.6)	Bacteremia	4 (1.9)
Dengue and Leptospirosis and Murine typhus	1 (0.6)	Scrub typhus	4 (1.9)
Dengue and Hepatitis A	1 (0.6)	Enteric fever	2 (0.9)
		Murine typhus and Influenza	2 (0.9)
		Bacteremia and influenza	1 (0.5)
		Leptospirosis and Influenza	1 (0.5)
		Leptospirosis and Scrub typhus	1 (0.5)
**Total**	**183**	**Total**	**214**

### Bayesian network model for dengue diagnosis

The model that only contained demographic characteristics, including age, underlying diseases, and occupation, provided an AUC value of only 0.65 for prediction of dengue infection. Clinical signs and symptoms, such as bleeding sign, duration of fever, petechial, myalgia, nausea, and tourniquet test result were factors that produced the best model for clinical manifestation with AUC of 0.72. When considering only the patient laboratory profile at diagnosis, the model that included white blood cell count, percent atypical lymphocyte, percent lymphocyte, hematocrit level, platelet count, and AST/ALT ratio provided the highest AUC of 0.87 (Table 4).

**Table 4:** Performance of different dengue diagnosis models, determined by Area Under the Curve (AUC) of ROC analysis.

Model	1	2	3	4	5	6	7
Demographic characteristics	✓			✓	✓	✓	✓
Clinical manifestations		✓		✓	✓	✓	✓
Laboratory indicators			✓	✓	✓	✓	✓
NS1 antigen test					✓		✓
Incidence rate						✓	✓
**AUC**	0.65	0.72	0.87	0.88	0.92	0.92	0.94

The performance of the dengue diagnostic model was improved when demographic, clinical manifestation, and laboratory indicators, were combined in the model, producing an AUC of 0.89. The AUC increased to 0.92 when the NS1 antigen test was added as one of the predictors. Introducing dengue incidence into the model improved the AUC by 0.03 (from 0.89 to 0.92) for the model without NS1 (model 6), and by 0.02 (from 0.92 to 0.94) for the model with NS1 (model 7) (Table 4). Therefore, model 6 and model 7 were considered as the final BN models, without and with the NS1 test, respectively.

### Accuracy of dengue diagnosis models

The diagnostic accuracy of BN models 6 and 7 was evaluated using 10-fold cross validation. Model 6 (without NS1) provided an AUC that ranged from 0.62 to 0.96, with average AUC of 0.749. For the model with NS1 (Model 7), 10-fold cross-validation provided an AUC that range from 0.72 to 0.97 with average AUC of 0.83 (Table 5).

**Table 5:** Mean and standard deviation of AUC using 10-fold cross-validation.

Testing data set (ID)	AUC Model 6 (without NS1)	AUC Model 7 (with NS1)
ID: 1–40	0.76	0.87
ID: 41–80	0.63	0.73
ID: 81–120	0.81	0.87
ID: 121–160	0.75	0.79
ID: 161–200	0.71	0.81
ID: 201–240	0.81	0.89
ID: 241–280	0.62	0.72
ID: 281–320	0.75	0.86
ID: 321–360	0.69	0.81
ID: 361–397	0.96	0.97
**Mean (SD)**	0.749 (0.09)	0.83 (0.07)

### Physician’s dengue diagnosis

There was moderate agreement of the dengue diagnosis made by the two physicians, with the Cohen’s Kappa coefficients of 0.59 (95% CI: 0.52–0.68) and 0.50 (95% CI: 0.42–0.59) for the diagnosis made from patient’s profile with and without NS1 rapid test result, respectively. For the case in which the two physicians disagreed, the final diagnosis was made by the third physician to represent the physician’s dengue diagnosis decision. The overall accuracy of the physician’s diagnosis was 77% and 74% when reviewing the patient’s profile with and without NS1 rapid test result, respectively. When the NS1 rapid test result was not available, the physicians could correctly detect 78% of the dengue cases (sensitivity); while about 71% of non-dengue cases were correctly detected (specificity) (Table 6). The NS1 rapid test result improved the specificity of the physician’s diagnosis to 79%, while the ability to detect dengue cases (sensitivity) reduced to 75% (Table 7).

**Table 6:** Overall accuracy of physician’s dengue diagnosis without NS1 rapid test result.

Physician’s diagnosis	Confirmed diagnosis		
	Dengue	Non-dengue	Total
**Dengue**	142	62	203
**Non-dengue**	41	152	193
**Total**	183	214	397

Sensitivity 78%; Specificity 71%

**Table 7:** Overall accuracy of physician’s dengue diagnosis with NS1 rapid test result.

Physician’s diagnosis	Confirmed diagnosis		
	Dengue	Non-dengue	Total
**Dengue**	137	44	181
**Non-dengue**	46	170	216
**Total**	183	214	397

Sensitivity 75%; Specificity 79%

### Comparison between expert and model performance

The overall performance in terms of accuracy of dengue diagnosis of the BN model and the physicians is shown in Table 8. For the BN model with the NS1 result, the sensitivity and specificity of the model was similar to that of the physicians (sensitivity 73.5% vs. 73.2%; specificity 78.8% vs. 79.4%, respectively), indicating that the model performance in making dengue diagnosis was comparable that of expert physicians (Table 8).

**Table 8:** Overall performance on dengue diagnosis of BN model and physician.

	Diagnosis result with NS1		Diagnosis result without NS1	
	Mean (95% CI)		Mean (95% CI)	
	Physician	BN model	Physician	BN model
**Sensitivity (%)**	73.2	73.5	76.3	73.5
	(66.9–79.5)	(63.7–83.7)	(68.9–83.7)	(62.5–84.5)
**Specificity (%)**	79.4	78.8	71.9	66.0
	(72.7–86.1)	(69.0–88.4)	(65.6–78.2)	(57.5–74.5)
**PPV (%)**	74.0	75.1	68.6	66.0
	(63.0–85.0)	(63.6–86.6)	(57.6–79.6)	(58.6–73.4)
**NPV (%)**	77.8	79.1	78.7	78.0
	(71.8–83.8)	(72.0–86.1)	(71.6–85.8)	(70.4–85.6)

Note: Mean and 95% Confidence Interval (CI) were calculated based on 10-fold cross validation datasets

When the NS1 rapid test result was not considered for diagnosis, the sensitivity of the physician diagnosis increased to 76.3%, but that of the model did not change. However, the specificity of the physicians and the model reduced to 71.9% and 66%, respectively. In the situation where the NS1 result is not available, the sensitivity and specificity of physician’s diagnosis were better than those of the model (Table 8).

## Discussion

Dengue is classified as one of many diseases in acute undifferentiated febrile illness. These diseases share similar characteristics of fever, and other clinical manifestations [8]. Although the WHO has recommended diagnostic criteria for dengue infection, physicians may still be uncertain in making diagnosis of dengue, particularly when laboratory confirmation is not available [23]. Machine learning techniques can be used to assist decision making in health practices. In this study, Bayesian networks were shown to be a promising technique to assist dengue diagnosis.

Bayesian networks can represent complex relationships among variables involved in a disease process. Previous studies have shown that Bayesian networks can be used to assist diagnosis in a variety of diseases, such as breast cancer, hepato-biliary diseases, liver disorder, pneumonia, and pressure ulcer [20, 24–27]. Our study also demonstrated that BN models can be effectively applied for dengue diagnosis. The BN model constructed in this study provided good diagnostic performance (AUC 0.75–0.80), even when considering only common clinical manifestations and basic laboratory tests. Our dengue diagnostic model was shown to perform as well as experts in infectious disease in terms of accuracy in diagnosing dengue using basic clinical and laboratory profile of patients. In areas where experienced physicians and laboratory resources are limited, the diagnostic model can be helpful to assist physician’s diagnosis of dengue.

In this study, variables involved in the BN were considered based on WHO recommendations for dengue diagnosis and statistical analysis of the data. Nausea, bleeding, petechial, myalgia, and tourniquet test were reported to have a relationship with dengue infection during the acute phase in many studies [1, 28–31]. In addition, lymphocyte, platelet, white blood cell count, AST/ALT, atypical lymphocyte, and hematocrit are also related to dengue infection. Interestingly, duration of fever prior to diagnosis appeared to influence physician diagnosis of dengue [1]. Clinical symptoms and laboratory results are usually dynamic over the course of illness [32–36]. Awareness of this can assist physicians in differentiating dengue patients from other patients. Therefore, our dengue diagnosis BN model also included duration of fever as prior information to determine the effect of clinical and laboratory profiles on the probability of having dengue. Individual background immunological profile for dengue or other flaviviruses due to direct infection or immunization may also determine clinical manifestation of current dengue infection. Since dengue vaccine is just recently available, including history of infection or immunization for dengue or other flavivirus diseases could potentially improve the performance of future BN diagnostic models.

The usefulness of the NS1 antigen rapid test for dengue diagnosis has been widely documented [37–40]. Although the WHO has recommended the NS1 rapid test as one of the diagnostic tests for dengue infection [1], the use of the test is still limited due to its high cost and low sensitivity [39]. In general, the result of the NS1 antigen rapid test should be carefully interpreted because its accuracy can change over the course of illness [39] following the dynamics of viral antigen and antibody levels [1]. Therefore, clinical information of patients must be considered along with the NS1 test result. Although 79% of patients in this study came to hospital within 5 days, the NS1 rapid test (dual test) alone showed 54% sensitivity (ability to correctly detect dengue cases) and 95% specificity (ability to correctly detect non-dengue cases). However, when considering patients’ clinical and laboratory information in the BN model, the sensitivity to detect dengue cases increased from 54% to 73% while the specificity to correctly identify non-dengue patients decreased but still maintained an acceptable level (from 95% to 79%).

Findings from this study suggest that including the NS1 rapid test result for clinical dengue diagnosis could improve the specificity both in the BN model and in physician’s decision, compared to the decision made with clinical and laboratory information alone. The specificity improved from about 70% to 79% when the NS1 result was included for decision making of both physician and BN model, whereas the sensitivity decreased from 76% to 73% for physician diagnosis when combined with NS1 results. These findings indicate that the NS1 rapid test would be useful to confirm non-dengue cases, but the test may not be helpful to improve the ability to detect dengue cases.

The model constructed in this study also included the monthly incidence of dengue infection in Bangkok reported by the Ministry of Public Health, Thailand. The incidence report is considered to be a useful source for prediction [41, 42]. Unfortunately, inclusion of incidence report data resulted in relatively small improvement of model performance in this study. Since the incidence was reported for the overall Bangkok area, this may not reflect the variation of dengue incidence in the specific geographical area where the patients were infected. A future study that includes incidence report data for more targeted and relevant population groups might be helpful to improve model performance.

The data used in this study was obtained from a cohort study of acute undifferentiated febrile patients at the Hospital for Tropical Diseases, Bangkok [21]. Data collected specifically for research purposes is likely to be more complete and valid, as compared with data generally collected for medical services. Laboratory confirmation for dengue, including PCR and serology tests, and other common tropical diseases were performed in all patients, which ensured the accuracy of our main outcome, dengue infection. However, using data from only one hospital may limit the generalizability of the results. As the Hospital for Tropical Diseases is a specialized hospital, patients who visited the hospital may be different from other hospitals in terms of disease distribution and patient characteristics. In this study, about 50% of AUFI patients had dengue infection. Whereas, a previous study conducted in Thailand in 2004 reported dengue infection in only 15% of AUFI patients [43]. Nevertheless, data collection in this study was conducted during 2013, when a dengue outbreak was reported throughout Thailand [44]. Moreover, the laboratory diagnosis of dengue in the previous study was based on only serology for dengue serotype 2 and 4 while the present study used both PCR and serology for all serotypes [21, 43]. These could explain the difference in proportion of dengue cases observed in the previous study. A large proportion of dengue infection among AUFI cases in this study can affect the model’s ability to correctly predict dengue infection when the model suggests positive for dengue (positive predictive value: PPV). In this study the PPV was 75% and 66% in the BN model with and without the NS1 test, respectively. These values could be lower if the model was applied in areas with a smaller proportion of dengue among AUFI cases. As a specialized hospital, acute febrile patients observed in this hospital may come with unusual manifestations that lead to difficulty in making diagnosis by inexperienced physicians. Therefore, data used in this study would benefit the usefulness of our BN model, in which the model could potentially assist decision making particularly in the difficult cases.

This study also compared the performance of the BN dengue diagnostic model with that of expert physicians. The results showed that the model performance is comparable with that of experts. Although the physician’s diagnosis was made according to the WHO criteria for dengue diagnosis, the agreement on dengue diagnosis among the two experts in this study was moderate with the Cohen’s Kappa coefficients of about 0.5. This suggests relatively low reliability of physician diagnosis [8, 12]. As expert performance in this study was determined by consensus of two or three physicians, the performance of other individual physicians may vary from that observed in this study. Use of a BN diagnostic model could help to increase reliability of the diagnosis. However, further study of implementation of the BN diagnostic model for prospective clinical diagnoses in different settings is required to confirm the validity and usefulness of the model in a real setting.

Accurate diagnosis of acute febrile illness is critical for proper management of patients. Lack of definite diagnosis may result in providing unnecessary empirical treatment. The BN diagnostic model developed in this study provides a high quality dengue diagnosis that can be used in low-resource settings with limited access to laboratory confirmation. The definitive diagnosis of dengue could permit administration of targeted treatment and withholding of empiric treatment or conservative observational hospital admission. It should be considered to increase dengue diagnostic performance and as a means of elevating diagnostic capacity for AFI in general. In addition, the speed and reliability of the BN model could support decision making in limited resource setting where physician fatigue is an issue since the performance of the BN model is independent of high volume of case load.

## Supporting information

S1 Checklist STROBE checklist. (DOCX)

S2 Checklist STARD checklist. (DOCX)
